# Identification and validation of pyroptosis-related genes as potential biomarkers for hypertrophic cardiomyopathy: A comprehensive bioinformatics analysis

**DOI:** 10.1097/MD.0000000000036799

**Published:** 2024-01-26

**Authors:** Xin Tang, Yi Shen, Yun Lu, Wanya He, Ying Nie, Xue Fang, Jinghui Cai, Xiaoyun Si, Yan Zhu

**Affiliations:** a School of Public Health, Guizhou Medical University, Guiyang, China; b Department of Cardiovascular Medicine, the Affiliated Hospital of Guizhou Medical University, Guiyang, China.

**Keywords:** bioinformatics, biomarkers, hypertrophic cardiomyopathy, pyroptosis-related genes

## Abstract

Pyroptosis plays a key role in the death of cells including cardiomyocytes, and it is associated with a variety of cardiovascular diseases. However, the role of pyroptosis-related genes (PRGs) in hypertrophic cardiomyopathy (HCM) is not well characterized. This study aimed to identify key biomarkers and explore the molecular mechanisms underlying the functions of the PRGs in HCM. The differentially expressed genes were identified by GEO2R, and the differentially expressed pyroptosis-related genes (DEPRGs) of HCM were identified by combining with PRGs. Enrichment analysis was performed using the “clusterProfiler” package of the R software. Protein-protein interactions (PPI) network analysis was performed using the STRING database, and hub genes were screened using cytoHubba. TF-miRNA coregulatory networks and protein-chemical interactions were analyzed using NetworkAnalyst. RT-PCR/WB was used for expression validation of HCM diagnostic markers. Quantitative reverse transcription-polymerase chain reaction (qRT-PCR) and Western Blot (WB) were used to measure and compare the expression of the identified genes in the cardiac hypertrophy model and the control group. A total of 20 DEPRGs were identified, which primarily showed enrichment for the positive regulation of cytokine production, regulation of response to biotic stimulus, tumor necrosis factor production, and other biological processes. These processes primarily involved pathways related to Renin-angiotensin system, Adipocytokine signaling pathway and NF-kappa B signaling pathway. Then, a PPI network was constructed, and 8 hub genes were identified. After verification analysis, the finally identified HCM-related diagnostic markers were upregulated gene protein tyrosine phosphatase non-receptor type 11 (*PTPN11*), downregulated genes interleukin-1 receptor-associated kinase 3 (*IRAK3)*, and annexin A2 (*ANXA2*). Further GSEA analysis revealed these 3 biomarkers primarily related to cardiac muscle contraction, hypertrophic cardiomyopathy, fatty acid degradation and ECM − receptor interaction. Moreover, we also elucidated the interaction network of these biomarkers with the miRNA network and known compounds, respectively. RT-PCR/WB results indicated that *PTPN11* expression was significantly increased, and *IRAK3* and *ANXA2* expressions were significantly decreased in HCM. This study identified *PTPN11, IRAK3*, and *ANXA2* as pyroptosis-associated biomarkers of HCM, with the potential to reveal the development and pathogenesis of HCM and could be potential therapeutic targets.

## 1. Introduction

Hypertrophic cardiomyopathy (HCM) is an inherited cardiovascular disease with left ventricular hypertrophy, myocardial hypercontraction, cardiomyocyte disturbance, and fibrosis.^[1]^ It is one of the most critical reasons behind sudden cardiac death in children and adolescents.^[2]^ Echocardiography shows that the incidence of HCM in adults is estimated to be 0.2%,^[3]^ and considering the clinical and genetic characteristics (including the patient family members), the prevalence reaches 0.5%.^[4]^ However, the pathophysiology of HCM is complex, and the clinical manifestations and prognosis are diverse, warranting successful diagnosis only when the cardiac morphology changes and function failure worsens. In addition, drug-mediated and surgical therapies are the most important treatment methods for HCM. However, these methods primarily play a role in reducing clinical symptoms, and to date, treatment plans for the subsequent developmental stages of the disease are lacking. Currently, the signaling pathways and regulatory networks underlying the pathogenesis of HCM are yet to be fully understood.^[5,6]^ Therefore, more in-depth exploration and identification of potential genes and biological pathways underlying the pathogenesis of HCM is crucial for diagnosis and treatment.

Pyroptosis, a novel form of programmed cell death, is characterized by cell swelling, numerous bubble-like protrusions, rupture of the plasma membrane, the release of cellular contents and inflammatory factors, and subsequent final activation of an inflammatory response.^[7]^ The balance between cell formation and death is essential for the cardiovascular system to maintain normal structure and function. Pyroptosis is a critical innate immune response in cardiovascular diseases. An increasing number of studies highlight the indispensable role of pyroptosis plays in various cardiovascular diseases, such as atherosclerosis, diabetic cardiomyopathy, and heart failure.^[8–10]^ Recent studies have also shown that the expression of pyroptosis-related genes is related to cardiac hypertrophy.^[11]^ However, no studies have yet investigated the mechanism of pyroptosis in HCM.

The development of high-throughput sequencing technology has enabled the understanding of diseases gene expression profiles in a more comprehensive and in-depth manner, which is helpful for precise diseases treatment. This study used high-throughput sequencing data to explore the mechanism underlying pyroptosis-related gene functions in HCM, providing new insights for the prevention and treatment of HCM.

## 2. Material and method

### 2.1. Data source

We downloaded the microarray expression profiling datasets GSE36961 and GSE89714 from the GEO database (https://www.ncbi.nlm.nih.gov/geo/). GSE36961 was analyzed based on the GPL15389 Illumina HumanHT-12 V3.0 expression beadchip platform, with 145 samples, including 106 HCM ones and 39 healthy controls. GSE89714 contained 5 HCM cases and 4 normal controls, on the GPL1115 Illumina HiSeq 2000 (Homo sapiens) platform. Since the data in this study are from public databases, there is no ethical approval involved.

### 2.2. Identifying differently expressed pyroptosis-related genes

The differentially expressed genes (DEGs) between HCM and healthy controls in GSE36961 were identified using GEO2R (https://www.ncbi.nlm.nih.gov/geo/geo2r/) with the inclusion criteria *P* < .05 and log2|FC|> 0.5. Three hundred eighty-eight pyroptosis-related genes were downloaded from the GeneCards database.^[12]^ Common genes between the DEGs and pyroptosis-related genes were identified as differentially expressed pyroptosis-related genes (DEPRGs). The“ggplot2” package in R was utilized to draw the volcano plots. Heatmap and PCA of DEPRGs were provided in this study by https://www.bioinformatics.com.cn, an online platform for data analysis and visualization.

### 2.3. GO, KEGG, and DO enrichment analyses of DEPRGs

Gene Ontology (GO) [biological process, cellular component, and molecular function (MF)], and the Kyoto Encyclopedia of Genes and Genomes (KEGG) functional enrichment analysis were performed using the “clusterProfiler” package of R software.^[13]^ The WebGestalt^[14]^ was used for DO enrichment analysis with *P* < .05 as the significance level.

### 2.4. Protein-protein interaction (PPI) network

We performed PPI network analysis using the STRING (https://string-db.org/) database to assess protein-protein interactions for DEPRGs. We mapped the DEPRGs onto the PPI network and set an interaction score > 0.4 as a threshold. In addition, we used the plug-in cytoHubba in Cytoscape v3.9.1 software to screen the top ten genes with the highest height values, respectively, by 4 algorithms (MCC, DMNC, MNC, and Degree) and visualized the overlapping hub genes via a Venn diagram (http://bioinformatics.psb.ugent.be/webtools/Venn/).

### 2.5. Validation of hub genes

The dataset GSE89714 contained 5 HCM and 4 healthy samples and was used as validation set to verify the expression of the hub genes. The statistically significant genes, as the key biomarkers, are instrumental in diagnosing and treating HCM.

### 2.6. Gene set enrichment analysis (GSEA)

According to the median gene expression, HCM samples were divided into high and low-expression groups. GSEA was performed to identify the significantly enriched relevant pathways.

### 2.7. TF-miRNA coregulatory network and Protein-chemical interactions

NetworkAnalyst^[15]^ (https://www.networkanalyst.ca/) was used to construct the TF-miRNA coregulatory network of biomarkers and identify their interacting compounds.

### 2.8. Cell culture

H9c2 cells were purchased from Procell Life Science and Technology Co., Ltd. (Wuhan, China). Cells were cultured in a complete DMEM medium containing 1 g/L glucose and 10% fetal bovine serum (FBS) in an incubator at 37 ° C with 5% CO_2_.

### 2.9. Cell viability measurement

Cell viability was determined by 3-(4,5)-dimethylthiahiazo(-z-y1)-2,5-di-phenytetrazoliumromide (MTT, Beijing Solarbio Science & Technology Co., Ltd.) assay. 1 × 10^4^ cells were seeded in 96-well plates in a volume of 100 μL per well. After 24 hours, the medium was discarded, and isoproterenol (Sigma–Aldrich, USA, 0, 2, 5, 10, and 20 μM, dissolved in DMEM containing 1% FBS) was added to the culture for 48 hours. The medium was replaced with a fresh one every 24 hours. The medium was discarded, and each well was washed twice with PBS. MTT (10 μL, 5mg/ml) solution was added to each well of the 96-well plate containing 90 μL medium. After 4 hours of incubation, the medium was discarded, and 150 μL dimethylsulfoxide from Solarbio was added to each well. The absorbance at 490 nm was measured to calculate the percentage of cell viability: Cell viability (%) = (OD_Iso_ − OD_blank_)/(OD _control_ − OD_blank_) × 100%.

### 2.10. Cell model establishment

The upregulation of ANP and β-MHC expression indicates that the cardiac hypertrophy model has been successfully induced. Cells (1 × 10^5^) in the logarithmic growth phase were seeded in each 35 mm cell culture dish. After 24 hours, cardiomyocytes were randomly divided into 2 groups: control group: given 2 ml DMEM containing 1% FBS for 48 hours; Iso group: given 10 μmol/L Iso in 2 ml DMEM containing 1% FBS for 48 hours. Medium for both groups was changed every 24 hours.

### 2.11. Real-time quantitative polymerase chain reaction (RT-PCR)

The mRNA levels of *ANP, β-MHC, IRAK3, PTPN11*, and *ANXA2* were detected by RT-PCR. Total RNA was extracted from cells using TRIzol reagent according to the manufacturer instructions. PrimeScript^TM^ RT reagent kit and TB Green premix (Takara Bio, USA) was used for cDNA synthesis and RT-PCR, respectively. The primer sequences are listed in Table 1. The RT-qPCR data were analyzed by the ΔΔCT method. *GAPDH* was used as an internal control for normalizing all gene expressions.

**Table 1:** qPCR primers.

Genes	Forward primer (5′-3′)	Reverse primer (5′-3′)
*ANP*	GGCACTTAGCTCCCTCTC	CCCTCAGTTTGCTTTTCA
*β-MHC*	TGGATGCAGACCTCTCCC	TGCTTCTTGCCACCCTTG
*IRAK3*	TGACACAGAAAACCCCCT	CCAGCATTCCTCACAAGA
*PTPN11*	GGAAACACAGAGAGAACC	TATCAATCACAATGAACG
*ANXA2*	GCAGTGTGTGCCACCTCC	GTCCCCTTGCCCTTCATG
*GAPDH*	GGCTCTCTGCTCCTCCCTGT	CGTTCACACCGACCTTCACC

### 2.12. Western blotting analysis

After treatment, the medium was removed, and the cells were washed with PBS. Cells were then lysed in cell lysis buffer containing 1% PMSF on ice for 30 minutes and centrifuged at 12 × 10^3^ rpm for 5 minutes. The total protein was quantified with the BCA method. The samples were separated on 10% SDS–PAGE gels and then transferred to polyvinylidene difluoride membranes. The membranes were blocked in 5% skim milk for 2 hours on a shaker and then incubated with primary antibodies such as β-Actin (Beyotime, China), IRAK3(Abcam, ab-8116), PTPN11 (CST, 3397S), ANXA2 (CST, 8235S) overnight at 4 °C. Membranes were washed with TBST and then incubated with corresponding HRP-conjugated secondary antibody for 1 hour at room temperature and detected by ECL detection kit (Bio-Rad, USA).

### 2.13. Statistical analysis

All data were expressed as mean ± standard deviation (SD). Statistical analysis and graphing were performed using GraphPad prism 9.0. One-way analysis of variance followed by Dunnett post hoc test was used to compare differences between multiple groups and *t* tests for the differences between the 2 groups. *P* < .05 was considered to be statistically significant.

## 3. Results

### 3.1. Identification of DEPRGs

**Figure 1.**
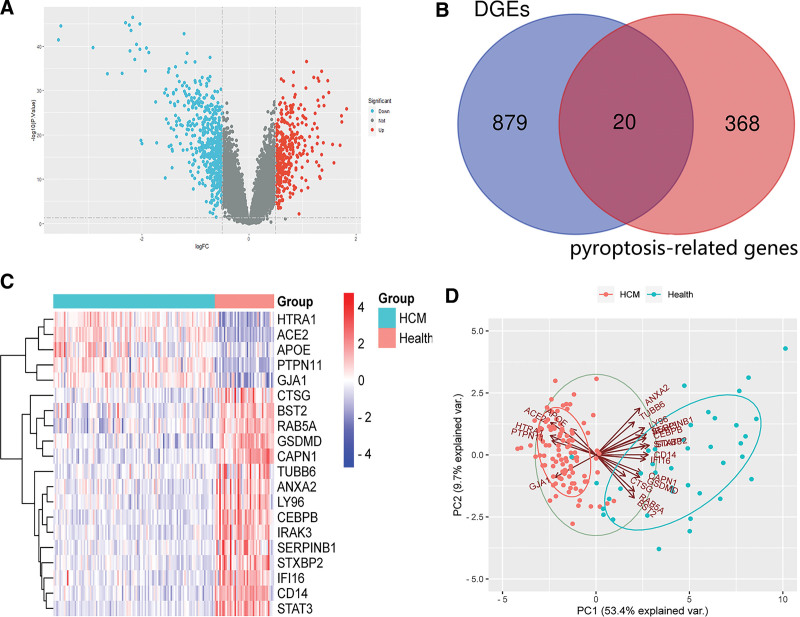
Identification of DEPRGs of HCM. (A) The differentially expressed genes of GSE36961. (B) The DEPRGs of HCM. (C) The heat map of the DEPRGs. (D) PCA plots. DEPRGs = differentially expressed pyroptosis-related genes, HCM = hypertrophic cardiomyopathy.

We used the dataset GSE36961 (log2 transformed and normalized) based on the inclusion criteria of |log2FC|>0.5, *P *< .05. This analysis revealed 899 DEGs (Fig. 1A), including 372 upregulated and 527 downregulated genes. Twenty congruous DEPRGs were identified by integrated bioinformatics analysis (Fig. 1B), including 5 upregulated and 15 downregulated genes, depicted by a heatmap (Fig. 1C). PCA analysis was performed on DEPRGs to clarify the good discrimination between HCM and healthy controls (Fig. 1D).

### 3.2. Function enrichment analyses of the DEPRGs

**Figure 2.**
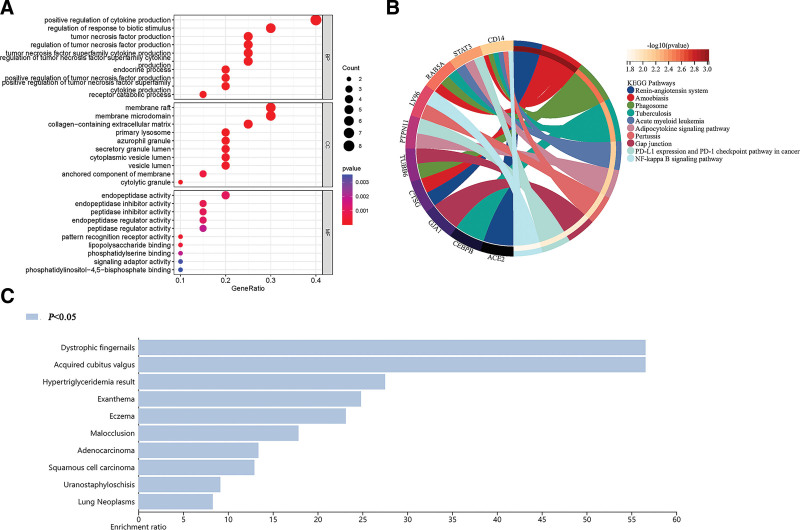
Function enrichment analyses of DEPRGs (*P* < .05). (A) GO; (B) KEGG signaling pathway; (C) DO enrichment. DEPRGs = differentially expressed pyroptosis-related genes, GO = Gene Ontology, KEGG = Kyoto Encyclopedia of Genes and Genomes.

The GO analysis of DEPRGs was performed to reveal their biological functions (Fig. 2A). BP analysis revealed how most DEPRGs were primarily involved in the positive regulation of cytokine production, regulation of response to biotic stimulus, tumor necrosis factor production. Cellular component analysis revealed that most DEPRGs were enriched into the membrane raft, membrane microdomain, and collagen-containing extracellular matrix. MF analysis revealed that the DEPRGs were significantly enriched in endopeptidase activity, peptidase inhibitor activity. KEGG enrichment analysis showed (Fig. 2B) that the DEPRGs were mainly functionally involved in pathways associated with Salmonella infection, Amoebiasis, Renin − angiotensin system, Adipocytokine signaling pathway and NF-kappa B signaling pathway. The WebGestalt online database was used for DO analysis to explore the function of DEPRGs further, which revealed that Dystrophic fingernails, Acquired cubitus valgus, Hypertriglyceridemia result, were the major diseases that the DEPRGs participated in (Fig. 2C).

### 3.3. PPI network analyses of DEPRGs

**Figure 3.**
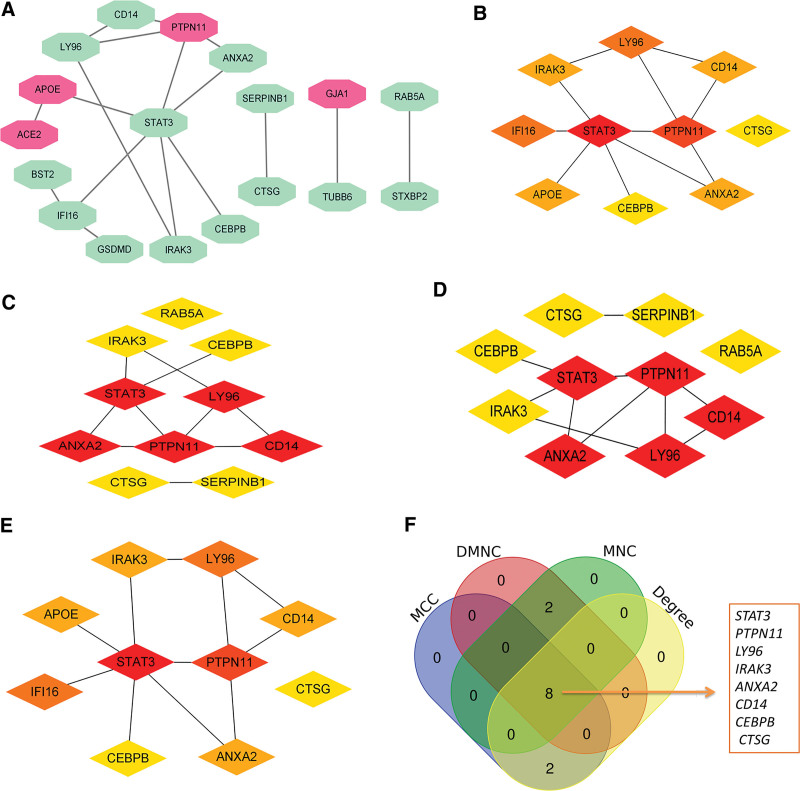
The PPI network analysis. (A) The PPI network of the DEPRGs. Red indicates upregulated, and green is downregulated. (B) Top 10 nodes detected by MCC. (C) Top 10 nodes detected by DMNC. (D) Top 10 nodes detected by MNC. (E) Top 10 nodes detected by Degree. (F) The 4 algorithms utilized to identify hub genes. DEPRGs = differentially expressed pyroptosis-related genes.

Based on the network interaction score > 0.4 according to the STRING database, a PPI network with 18 nodes and 17 edges was built. Moreover, among the 18 nodes, 4 nodes were upregulated, and 14 were downregulated (Fig. 3A). The top ten genes were detected via 4 algorithms [MCC (Fig. 3B), DMNC (Fig. 3C), MNC (Fig. 3D), and Degree (Fig. 3E)], respectively, of cytoHubba plug-in in Cytoscape. Finally, the 8 common genes by the 4 algorithms were identified as the hub genes of HCM: one upregulated gene protein tyrosine phosphatase non-receptor type 11 (*PTPN11*), 7 downregulated genes signal transducer and activator of transcription 3 (*STAT3*), lymphocyte antigen 96 (*LY96*), interleukin-1 receptor-associated kinase 3 (*IRAK3*), annexin A2 (*ANXA2*), CD14 molecule (*CD14*), CCAAT enhancer binding protein beta *(CEBPB)*, and cathepsin G (*CTSG*) (Fig. 3F).

### 3.4. Gender differences in HCM

We analyzed the GSE36961 dataset with 145 samples, including 19 healthy men and 54 male HCM patients, and 20 healthy women and 52 female HCM patients, to explore whether there are gender differences in the prevalence of HCM. The difference was not statistically significant (χ^2^ = 0.056, *P* = .812), suggesting that the prevalence of HCM does not differ between men and women. In addition, we selected HCM patient samples from this dataset, performed differential expression analysis by gender, and found no gender differences in the expression of these 8 hub genes in HCM patients (Table 2).

**Table 2:** Eight hub genes in HCM patients grouped by gender.

Gene symbol	adj.P.Val	*P* value	logFC
*PTPN11*	0.954	.213	0.073815
*IRAK3*	0.965	.268	−0.04424
*ANXA2*	0.981	.363	−0.06047
*STAT3*	0.972	.322	−0.05204
*LY96*	0.997	.606	−0.03469
*CD14*	0.999	.826	−0.01925
*CEBPB*	0.899	.0903	0.093459
*CTSG*	0.999	.64	0.031557

### 3.5. Verification of the hub genes for HCM

**Figure 4.**
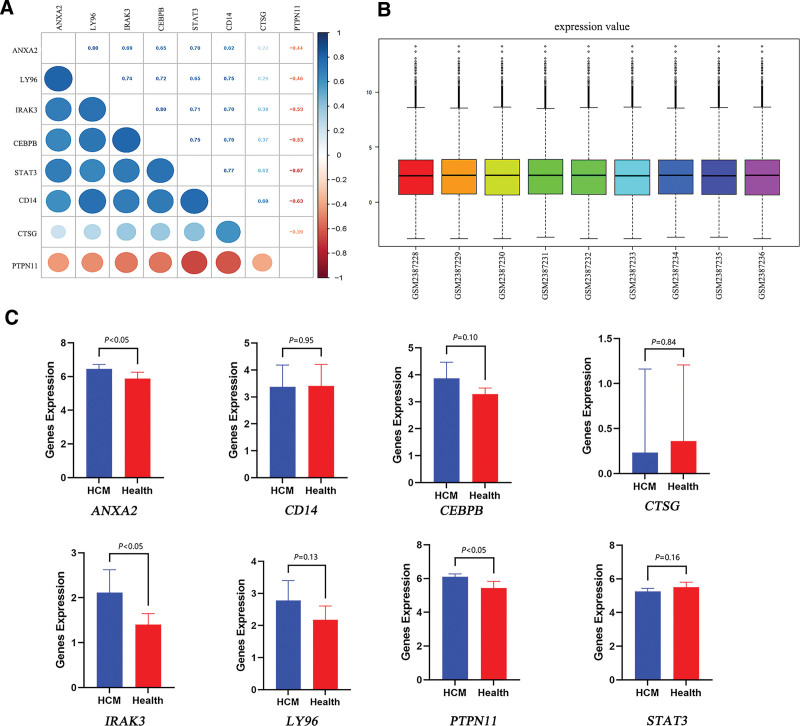
Validation of hub genes. (A) Heat-map of the correlation analysis between candidate genes. (B) Normalization of GSE89714. (C) Data validation of hub genes by GSE89714.

We then performed a correlation analysis on the 8 hub genes to verify their correlation and improve the diagnostic value (Fig. 4A), which revealed a significant association with each other except *CTSG*, indicating significant shared functional similarities. Analyzing the normalized dataset GSE89714 (Fig. 4B), revealed that only *PTPN11, IRAK3*, and *ANXA2* expressions were statistically significant between the 2 groups, with the expression of *PTPN11, IRAK3*, and *ANXA2* in HCM being higher than that in the healthy group (Fig. 4C). The expression of *PTPN11* was consistent with GSE36961, while that of *IRAK3* and *ANXA2* were opposite. Therefore, further studies are needed to validate the expression of the biomarkers. In short, our results indicated the potential significance of *PTPN11, IRAK3*, and *ANXA2* as essential biomarkers associated with pyroptosis in HCM.

### 3.6. GSEA of PTPN11, IRAK3, and ANXA2

**Figure 5.**
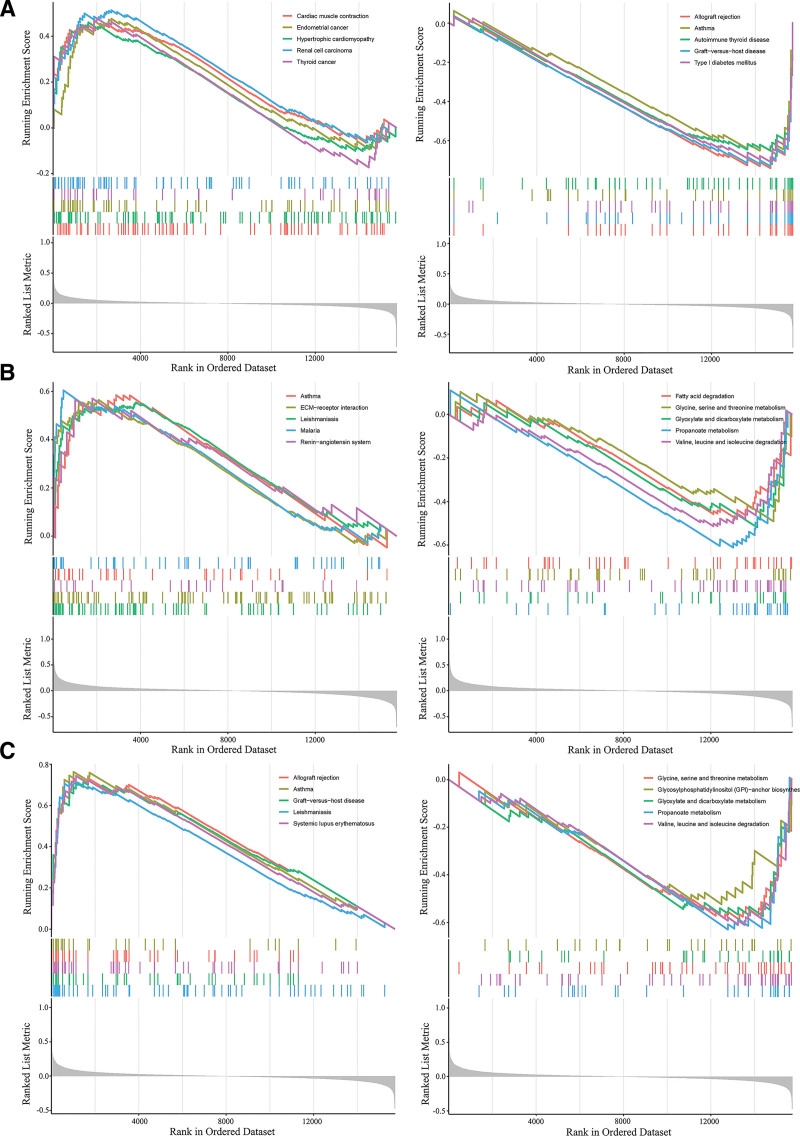
Gene set enrichment analysis. (A) GSEA results of *PTPN11*. (B) GSEA results of *ANXA2*. (C) GSEA results of *IRAK3*.

Taking a cue from above analysis, we chose *PTPN11, ANXA2*, and *IRAK3* for further analysis. GSEA showed that *PTPN11* was functionally involved in Asthma, Autoimmune thyroid disease, Cardiac muscle contraction, and Hypertrophic cardiomyopathy, indicating its direct connection with HCM as a critical biomarker (Fig. 5A). *ANXA2* and *IRAK3* were closely related to the glycine, serine, and threonine metabolism, glyoxylate and dicarboxylate metabolism, Leishmaniasis, propanoate metabolism, valine, leucine, and isoleucine degradation. In addition, *ANXA2* was also associated with ECM − receptor interaction, fatty acid degradation, and the renin − angiotensin system (Fig. 5B and C).

### 3.7. TF-miRNA coregulatory network and protein-chemical interactions

**Figure 6.**
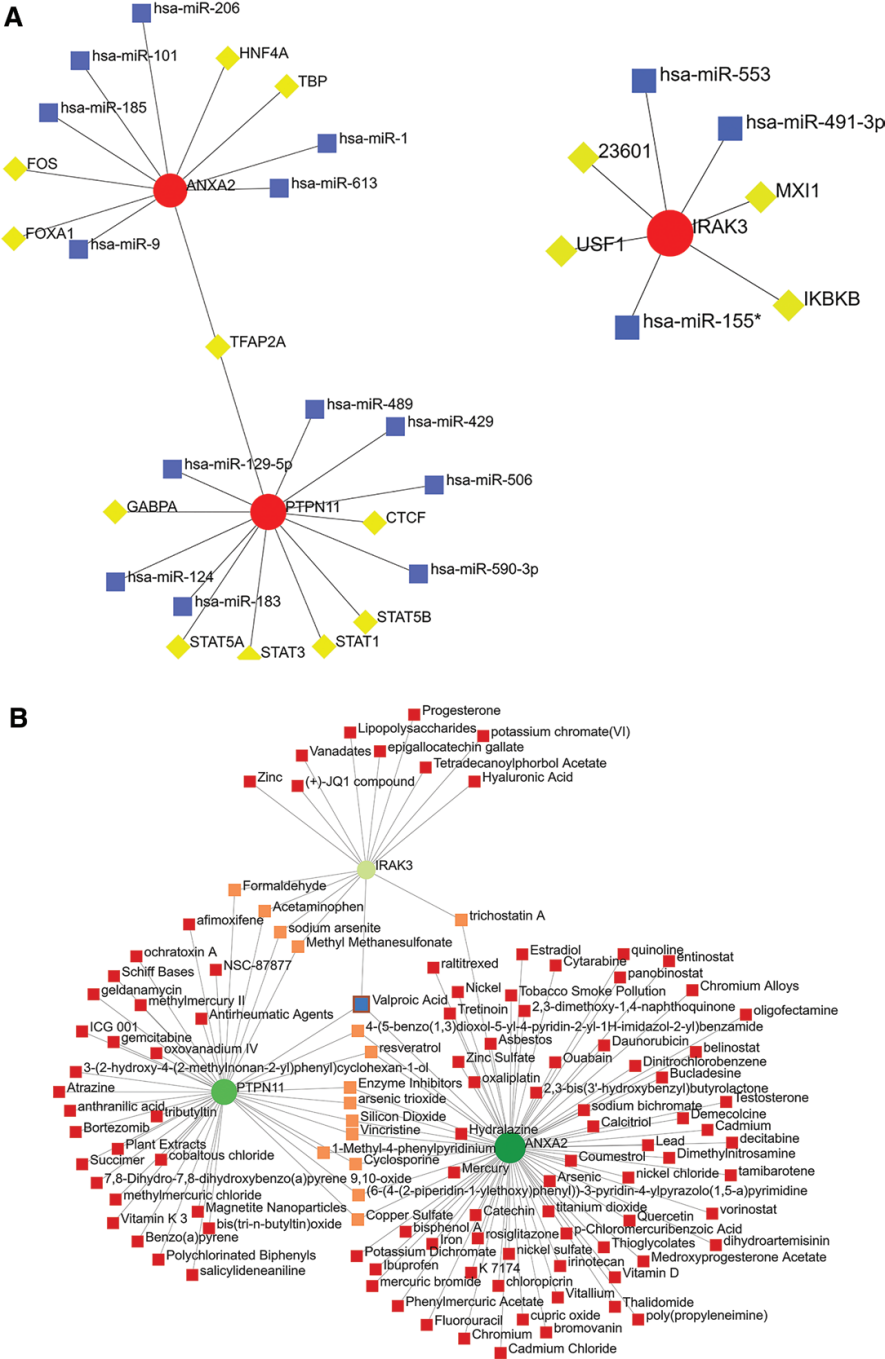
NetworkAnalyst analysis of hub genes. (A) TF-miRNA coregulatory network. The red nodes represent the key biomarkers, the yellow nodes are TF genes, and the blue nodes indicate miRNA. (B) Protein-chemical interactions. The green nodes represent the key biomarkers. The pink nodes represent compounds that interact in common between 2 key biomarkers. The blue node represent compound that interact with the 3 key biomarkers.

TF-miRNA coregulatory network showed the interaction of TF genes and miRNAs with the 3 key biomarkers (Fig. 6A). *PTPN11* and *ANXA2* were co-regulated by transcription factor AP-2 alpha (TFAP2A). In addition, we identified a compound, Valproic Acid, that could interact with the 3 key biomarkers (Fig. 6B). Since these compounds were detected against 3 key biomarkers, they represented common compounds in HCM. The compounds interacting with most key biomarkers are listed in Table 3.

**Table 3:** Top 16 compounds with key biomarker interactions.

Id	Label	Degree	Betweenness	Gene
D014635	Valproic Acid	3	602.42	*PTPN11/IRAK3/ANXA2*
C012589	trichostatin A	2	353.98	*IRAK3/ANXA2*
D015655	1-Methyl-4-phenylpyridinium	2	178.47	*IRAK3/ANXA2*
C459179	4-(5-benzo(1,3)dioxol-5-yl-4-pyridin-2-yl-1H-imidazol-2-yl)benzamide	2	178.47	*IRAK3/ANXA2*
C516138	(6-(4-(2-piperidin-1-ylethoxy)phenyl))-3-pyridin-4-ylpyrazolo(1,5-a)pyrimidine	2	178.47	*IRAK3/ANXA2*
C006632	arsenic trioxide	2	178.47	*IRAK3/ANXA2*
D019327	Copper Sulfate	2	178.47	*IRAK3/ANXA2*
D016572	Cyclosporine	2	178.47	*IRAK3/ANXA2*
D004791	Enzyme Inhibitors	2	178.47	*IRAK3/ANXA2*
C059514	resveratrol	2	178.47	*IRAK3/ANXA2*
D012822	Silicon Dioxide	2	178.47	*IRAK3/ANXA2*
D014750	Vincristine	2	178.47	*IRAK3/ANXA2*
D000082	Acetaminophen	2	69.97	*PTPN11/ANXA2*
D005557	Formaldehyde	2	69.97	*PTPN11/ANXA2*
D008741	Methyl Methanesulfonate	2	69.97	*PTPN11/ANXA2*
C017947	sodium arsenite	2	69.97	*PTPN11/ANXA2*

### 3.8. Effect of isoproterenol on cardiomyocytes viability

**Figure 7.**
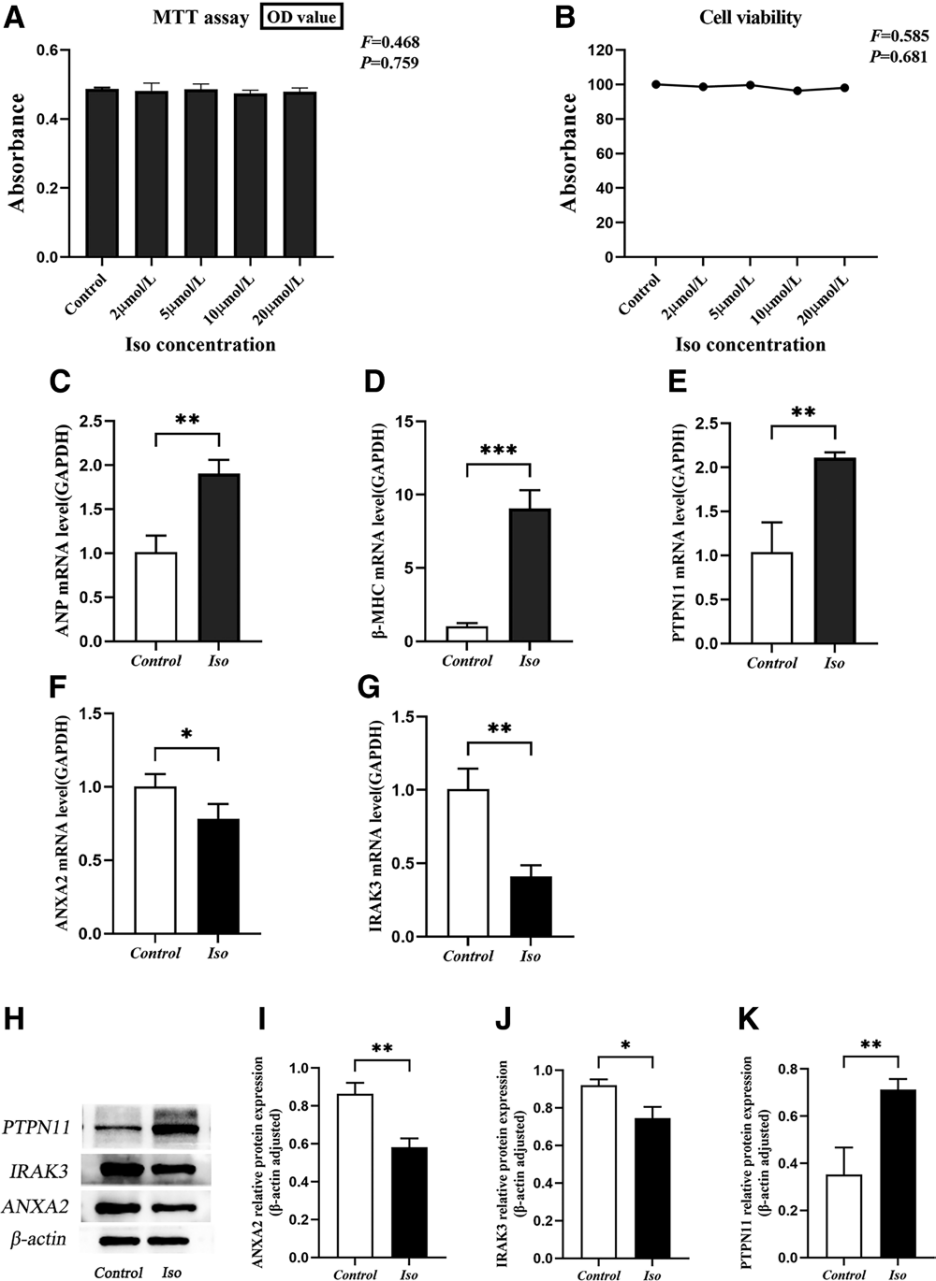
The expression levels of PTPN11, IRAK3, and ANXA2 in isoproterenol-induced H9c2 cardiomyocytes. (A) Variations in absorbance values of cells treated with different concentrations of Iso. (B) Iso-treated cell viability. The viability of the control group was considered 100%, and the viability was measured by MTT assay. (C–G) mRNA levels of ANP, β-MHC, PTPN11, ANXA2, IRAK3 in isoproterenol induced H9c2 cardiomyocytes. (H) Representative western blots showing PTPN11, IRAK3, and ANXA2 levels. (I–K) Quantification of the relative changes in PTPN11, IRAK3, and ANXA2 expression. *P < .05, **P < .01 compared with Control group. All values are mean ± SD. MTT = 3-(4,5)-dimethylthiahiazo(-z-y1)-2,5-di-phenytetrazoliumromide.

Next, we incubated the cells with different concentrations of Iso (0, 2, 5, 10, 20 μmol/L) for 48 hours to determine its cytotoxic effects. Generally, OD values between 0 and 7 indicate that the linear relationship of cell viability is consistent. In this study, the OD values corroborated this notion (Fig. 7A). Assuming that the cell viability of the control group was 100% (0 μmol/L), the MTT assay showed that the concentrations of isoproterenol from 2 to 20 μmol/L failed to significantly affect the viability of H9c2 cells (Fig. 7B). Therefore, gene expression changes in subsequent studies ruled out the cause of cell viability or toxicity. Based on previous studies^[16]^ and our results, 10 μmol/L Iso was chosen to induce H9c2 cardiac hypertrophy.

### 3.9. The mRNA expression of hypertrophy-related fetal genes and pyroptosis-related genes in isoproterenol-induced H9c2 cardiomyocytes

The mRNA expression of *ANP* and *β-MHC* was significantly increased in the Iso (10 µmol/L) group compared to the control group (*P* < .05), indicating the successful establishment of the cardiac hypertrophy model (Fig. 7C–G). In addition, the mRNA expression of *PTPN11* was significantly higher in HCM than control (*P* < .05), and the mRNA expression of *ANXA2* and *IRAK3* was lower than control (*P* < .05).

### 3.10. The protein expression of *PTPN11, IRAK3*, and *ANXA2* induced by isoproterenol

The protein expression of *PTPN11* was significantly increased in the Iso group (*P* < .05). However, compared with the control group, Iso-induced *ANXA2* and *IRAK3* protein expression were decreased (*P* < .05) (Fig. 7H–K).

## 4. Discussion

This study identified 20 DEPRGs, and the molecular mechanisms underlying the functions of these genes in hypertrophic cardiomyopathy were further explored. GO analysis revealed a robust association of the DEPRGs to positive regulation of cytokine production, regulation of response to biotic stimulus, tumor necrosis factor production, endocrine process receptor, catabolic process, and other biological processes. We hypothesized that these genes might play a key role in HCM pathogenesis by regulating these biological processes. Increasing evidence shows an essential role of proinflammatory cytokines in the treatment and prognosis of cardiovascular diseases.^[17]^ Cytokines produced from different sources may also be of varying importance.^[18]^ The production of tumor necrosis factors also stimulates cardiac hypertrophy.^[19]^ Moreover, the regulation of catabolic processes plays a vital role in the pathophysiology of HCM.^[20]^ On the other hand, our study found that most of these DEPRGs were enriched in the Salmonella infection, Amoebiasis, Renin − angiotensin system, and other related signaling pathways. Previous studies have shown that salmonella infection might lead to left ventricular hypertrophy, causing cardiovascular complications.^[21,22]^ Recent studies have shown that the Renin-angiotensin system plays an essential role in the etiology of cardiomyopathy and can be used as a therapeutic target.^[23,24]^ In addition, PPI network analysis identified 8 hub genes*-STAT3, PTPN11, LY96, IRAK3, ANXA2, CD14, CEBPB*, and *CTSG*. Validation analysis of GSE89714 revealed *PTPN11, ANXA2*, and *IRAK3*, with statistically significant differential expression, as potential biomarkers of HCM. Furthermore, several compounds interacting with the biomarkers have been identified by NetworkAnalyst. For example, histone deacetylase inhibitor Trichostatin A and valproic acid attenuate cardiac hypertrophy,^[25]^ potentially contributing to the prevention and treatment of HCM.

*PTPN11*, encoding SHP2 protein, is a classic non-receptor protein tyrosine phosphatase, an essential participant in cell signal transduction, regulating cell growth and proliferation.^[26]^ Aberrant expression of *PTPN11* can lead to various diseases, for example, *PTPN11* is the most common mutant gene in Noonan syndrome (NS) and LEOPARD syndrome (LS),^[27]^ HCM being one of the characteristics of these 2 diseases. This phenomenon indicates some connection between *PTPN11* and HCM. Our GESA results indicated that a high expression of *PTPN11* might activate myocardial contraction and hypertrophic cardiomyopathy. Many studies also showed that *PTPN11* mutations were a common cause of RASopathy-associated HCM.^[28,29]^ Genotypic-phenotypic correlation studies showed that 8% of SHP2-related NS patients had HCM,^[30]^ and about 70% to 80% of LS cases manifested HCM.^[31]^ The main reason for RASopathy-associated HCM includes *PTPN11* mutation leading to abnormal signal transduction and cardiac hypertrophy during cardiac development.^[32]^ Previous studies have shown RAS-mitogen activated protein kinase signal transduction pathway and its upstream and downstream pathways to be upregulated in HCM,^[33]^ wherein *PTPN11* mutations critically induce cardiac hypertrophy, suggesting an effective treatment for HCM by blocking this signal pathway.^[34]^ Moreover, our study found a higher expression of *PTPN11* in HCM than in the healthy group and demonstrated that the gene was highly expressed in the cardiac hypertrophy model. A recent review elucidated how the overexpression of SHP2 is associated with hereditary developmental diseases.^[35]^ Therefore, we speculated a strong correlation between HCM development and the overexpression of *PTPN11*. In addition, many studies have highlighted how SHP2 inhibitors play an essential role in cancer treatment. The combination of SHP2 allosteric inhibitors and targeted molecular drugs is a promising therapeutic strategy for cancer.^[36]^ However, whether SHP2 inhibitors help treat HCM or not warrants further research.

*ANXA2*, a Ca^2+^-binding multifunctional protein, plays an essential role in various signal pathways and is found in various human tissues and cell types.^[37]^ *ANXA2* is associated with cardiovascular diseases; it is overexpressed in heart failure, for which it can be used as a candidate biomarker.^[38]^ Lower *ANXA2* expression increases plasma levels of proprotein converting enzyme subtilisin/ kexin 9, increasing the low-density lipoprotein cholesterol levels and the risk of coronary heart disease.^[39]^ Coincidentally, low-density lipoprotein cholesterol is also a prognostic biomarker for HCM.^[40]^ Interestingly, *ANXA2* showed a lower expression in the cardiac hypertrophy model, indicating that regulating its expression might reduce the condition and improve cardiac function. Moreover, GSEA results showed that lower expression of *ANXA2* protein might inhibit fatty acid degradation, glycine, serine, and threonine metabolism, glyoxylic acid, and dicarboxylic acid metabolism pathways. It has been demonstrated that glycine, serine, and threonine metabolic pathways are differentially regulated in HCM,^[41]^ and targeted amino acid metabolomics can accurately identify HCM.^[42]^ Glycine promotes the biosynthesis of the antioxidant glutathione, reduces mitochondrial oxygen production, and increases adenosine triphosphate production, leading to improved heart function and ventricular remodeling.^[43]^ The protein kinase C (PKC) family of serine/threonine protein kinases has attracted much attention for their roles in cardiac function, and one of its principal members, protein kinase C alpha (PKCα), critically mediates cardiac hypertrophy by regulating kinase 1/2 (ERK-1/2) through extracellular signaling.^[44,45]^ Moreover, the oxidative capacity of fatty acids is known to regulate cardiac hypertrophy.^[46]^ The regulation of the expression of genes related to fatty acid metabolism alleviates cardiac dysfunction and improves cardiac hypertrophy, potentially mediated by activating the sirtuin 3/AMP-activated protein kinase pathway.^[47]^ This suggests that fatty acid degradation may also be a potential mechanism to be therapeutically targeted for HCM treatment. In addition, this study indicated that the lower expression of *ANXA2* protein might also activate asthma, ECM-receptor interaction, leishmaniasis, malaria, and renin-angiotensin system (RAS) pathways, all of which might potentially regulate HCM treatment. RAS balances blood pressure and body fluids. Abnormal activation of this system produces the major bioactive hormone angiotensin (Ang) II, which binds to specific receptors and triggers a wide range of biological effects.^[48]^ In this regard, Ang II has been widely used to induce left ventricular hypertrophy,^[49,50]^ and the antagonists of the renin-angiotensin system have been reported to help treat HCM.^[51]^ More importantly, a growing number of studies discussed that activation of the renin-angiotensin-aldosterone system exacerbates left ventricular hypertrophy through increased blood pressure and the direct effects of the active peptides angiotensin-II (AT-II) and aldosterone on cardiomyocytes.^[52,53]^ Renin-angiotensin-aldosterone system blockade has been implicated in having potential therapeutic benefits in patients with HCM.^[54]^ Additionally, ECM-receptor interaction is an essential pathway in cardiac hypertrophy.^[55,56]^ Therefore, we hypothesized that ANXA2 might regulate a potential mechanism that could serve as a therapeutic target for HCM.

*IRAK3* is a member of the interleukin-1 receptor-associated kinase (IRAK) family, primarily in monocytes and macrophages, which led to it being named IRAK-M.^[57]^ IRAK-M is a negative regulator of Toll-like receptor signaling and critically regulates inflammation in the innate immune system.^[58]^ Some studies have shown that Nuclear factor-kappaB (NF-kB) activity or the level of inflammatory cytokines such as tumor necrosis factor-α and interleukin-6 (IL-6) increased upon *IRAK3* downregulation, indicating how it inhibits excessive inflammatory cytokine production in inflammation.^[59,60]^ NF-kB is a transcription factor that regulates gene expression and regulates inflammation, cell proliferation, and apoptosis.^[61]^ A large number of studies elucidated how the abnormal activation of NF-kB and increased levels of proinflammatory cytokines are involved in the regulatory mechanisms of cardiac hypertrophy,^[62,63]^ and ten years later, the recent studies have evaluated the correlation between NF-kB and the disease state of HCM patients.^[64]^ Myocardial fibrosis in HCM is caused by an inflammatory response.^[65]^ Moreover, tumor necrosis factor-α and IL-6 also play an essential role in the pathogenesis of cardiac hypertrophy.^[66,67]^ Also, reducing inflammatory cytokines is beneficial in attenuating cardiac hypertrophy. Our GSEA results showed that lower expression of *IRAK3* inhibits glycine, serine, and threonine metabolism pathway, further demonstrating the importance of *IRAK3* in HCM. Therefore, targeting *IRAK3* may contribute to developing novel therapeutic drugs for HCM.

## 5. Conclusion

In summary, this study identified *PTPN11, ANXA2*, and *IRAK3* as essential novel biomarkers of HCM associated with pyroptosis. We also screened the related pathways and compounds, which would play a crucial role in delineating the development and pathogenesis of HCM and provide potential therapeutic targets.

## Author contributions

**Methodology:** Xin Tang.

**Supervision:** Yan Zhu.

**Writing – original draft:** Xin Tang.

**Writing – review & editing:** Xin Tang, Yi Shen, Yun Lu, Wanya He, Ying Nie, Xue Fang, Jinghui Cai, Xiaoyun Si, Yan Zhu.

## References

[1] Tuohy CV Kaul S Song HK. Hypertrophic cardiomyopathy: the future of treatment. Eur J Heart Fail. 2020;22:228–40. 31919938 10.1002/ejhf.1715

[2] Alexander PMA Nugent AW Daubeney PEF. Long-term outcomes of hypertrophic cardiomyopathy diagnosed during childhood: results from a national population-based study. Circulation. 2018;138:29–36. 29490994 10.1161/CIRCULATIONAHA.117.028895

[3] Sen-Chowdhry S Jacoby D Moon JC. Update on hypertrophic cardiomyopathy and a guide to the guidelines. Nat Rev Cardiol. 2016;13:651–75. 27681577 10.1038/nrcardio.2016.140

[4] Semsarian C Ingles J Maron MS. New perspectives on the prevalence of hypertrophic cardiomyopathy. J Am Coll Cardiol. 2015;65:1249–54. 25814232 10.1016/j.jacc.2015.01.019

[5] Walsh R Buchan R Wilk A. Defining the genetic architecture of hypertrophic cardiomyopathy: re-evaluating the role of non-sarcomeric genes. Eur Heart J. 2017;38:3461–8. 28082330 10.1093/eurheartj/ehw603 PMC5837460

[6] Maron BJ Maron MS Maron BA. Moving beyond the sarcomere to explain heterogeneity in hypertrophic cardiomyopathy: JACC review topic of the week. J Am Coll Cardiol. 2019;73:1978–86. 31000001 10.1016/j.jacc.2019.01.061 PMC6550351

[7] Tan Y Chen Q Li X. Pyroptosis: a new paradigm of cell death for fighting against cancer. J Exp Clin Cancer Res. 2021;40:153. 33941231 10.1186/s13046-021-01959-x PMC8091792

[8] Qian Z Zhao Y Wan C. Pyroptosis in the initiation and progression of atherosclerosis. Front Pharmacol. 2021;12:652963. 34122076 10.3389/fphar.2021.652963 PMC8187899

[9] Lu Y Lu Y Meng J. Pyroptosis and its regulation in diabetic cardiomyopathy. Front Physiol. 2022;12:791848. 35145423 10.3389/fphys.2021.791848 PMC8822267

[10] Chai R Xue W Shi S. Cardiac remodeling in heart failure: role of pyroptosis and its therapeutic implications. Front Cardiovasc Med. 2022;9:870924. 35509275 10.3389/fcvm.2022.870924 PMC9058112

[11] Dai F Li X Li X. Caspase-1 abrogates the salutary effects of hypertrophic preconditioning in pressure overload hearts via IL-1β and IL-18. Front Mol Biosci. 2021;8:641585. 33842546 10.3389/fmolb.2021.641585 PMC8024560

[12] Stelzer G Rosen N Plaschkes I. The GeneCards suite: from gene data mining to disease genome sequence analyses. Curr Protoc Bioinformatics. 2016;54:1.30.1–1.30.33. 10.1002/cpbi.5 27322403

[13] Yu G Wang LG Han Y. clusterProfiler: an R package for comparing biological themes among gene clusters. OMICS. 2012;16:284–7. 22455463 10.1089/omi.2011.0118 PMC3339379

[14] Liao Y Wang J Jaehnig EJ. WebGestalt 2019: gene set analysis toolkit with revamped UIs and APIs. Nucleic Acids Res. 2019;47:W199–205. 31114916 10.1093/nar/gkz401 PMC6602449

[15] Zhou G Soufan O Ewald J. NetworkAnalyst 3.0: a visual analytics platform for comprehensive gene expression profiling and meta-analysis. Nucleic Acids Res. 2019;47:W234–41. 30931480 10.1093/nar/gkz240 PMC6602507

[16] Shang L Pin L Zhu S. Plantamajoside attenuates isoproterenol-induced cardiac hypertrophy associated with the HDAC2 and AKT/ GSK-3β signaling pathway. Chem Biol Interact. 2019;307:21–8. 31009642 10.1016/j.cbi.2019.04.024

[17] Arkoumani M Papadopoulou-Marketou N Nicolaides NC. The clinical impact of growth differentiation factor-15 in heart disease: a 2019 update. Crit Rev Clin Lab Sci. 2020;57:114–25. 31663791 10.1080/10408363.2019.1678565

[18] Mándi Y Hogye M Talha EM. Cytokine production and antibodies against heat shock protein 60 in cardiomyopathies of different origins. Pathobiology. 2000;68:150–8. 11174073 10.1159/000055916

[19] Ma ZG Yuan YP Zhang X. C1q-tumour necrosis factor-related protein-3 exacerbates cardiac hypertrophy in mice. Cardiovasc Res. 2019;115:1067–77. 30407523 10.1093/cvr/cvy279

[20] Ranjbarvaziri S Kooiker KB Ellenberger M. Altered cardiac energetics and mitochondrial dysfunction in hypertrophic cardiomyopathy. Circulation. 2021;144:1714–31. 34672721 10.1161/CIRCULATIONAHA.121.053575 PMC8608736

[21] Mokhobo KP. Typhoid cardiac involvement. S Afr Med J. 1975;49:55–6. 123083

[22] Filip C Nicolescu A Cinteza E. Cardiovascular complications of hemolytic uremic syndrome in children. Maedica (Bucur). 2020;15:305–9. 33312244 10.26574/maedica.2020.15.3.305 PMC7726508

[23] Rani B Kumar A Bahl A. Renin-angiotensin system gene polymorphisms as potential modifiers of hypertrophic and dilated cardiomyopathy phenotypes. Mol Cell Biochem. 2017;427:1–11. 28120210 10.1007/s11010-016-2891-y

[24] Onoue K Saito Y. New treatment for myocardial diseases. J Cardiol. 2021;77:620–5. 33250269 10.1016/j.jjcc.2020.10.016

[25] Kee HJ Sohn IS Nam KI. Inhibition of histone deacetylation blocks cardiac hypertrophy induced by angiotensin II infusion and aortic banding. Circulation. 2006;113:51–9. 16380549 10.1161/CIRCULATIONAHA.105.559724

[26] Cohen P. The regulation of protein function by multisite phosphorylation--a 25 year update. Trends Biochem Sci. 2000;25:596–601. 11116185 10.1016/s0968-0004(00)01712-6

[27] Tartaglia M Kalidas K Shaw A. PTPN11 mutations in Noonan syndrome: molecular spectrum, genotype-phenotype correlation, and phenotypic heterogeneity. Am J Hum Genet. 2002;70:1555–63. 11992261 10.1086/340847 PMC379142

[28] Chen H Li X Liu X. Clinical and mutation profile of pediatric patients with RASopathy-associated hypertrophic cardiomyopathy: results from a Chinese cohort. Orphanet J Rare Dis. 2019;14:29. 30732632 10.1186/s13023-019-1010-z PMC6367752

[29] Wang J Chandrasekhar V Abbadessa G. In vivo efficacy of the AKT inhibitor ARQ 092 in Noonan Syndrome with multiple lentigines-associated hypertrophic cardiomyopathy. PLoS One. 2017;12:e0178905. 28582432 10.1371/journal.pone.0178905 PMC5459472

[30] Sznajer Y Keren B Baumann C. The spectrum of cardiac anomalies in Noonan syndrome as a result of mutations in the PTPN11 gene. Pediatrics. 2007;119:e1325–31. 17515436 10.1542/peds.2006-0211

[31] Limongelli G Pacileo G Marino B. Prevalence and clinical significance of cardiovascular abnormalities in patients with the LEOPARD syndrome. Am J Cardiol. 2007;100:736–41. 17697839 10.1016/j.amjcard.2007.03.093

[32] Lauriol J Cabrera JR Roy A. Developmental SHP2 dysfunction underlies cardiac hypertrophy in Noonan syndrome with multiple lentigines. J Clin Invest. 2016;126:2989–3005. 27348588 10.1172/JCI80396 PMC4966304

[33] Shimada YJ Raita Y Liang LW. Comprehensive proteomics profiling reveals circulating biomarkers of hypertrophic cardiomyopathy. Circ Heart Fail. 2021;14:e007849. 34192899 10.1161/CIRCHEARTFAILURE.120.007849 PMC8292216

[34] Gelb BD Tartaglia M. RAS signaling pathway mutations and hypertrophic cardiomyopathy: getting into and out of the thick of it. J Clin Invest. 2011;121:844–7. 21339640 10.1172/JCI46399 PMC3046639

[35] Yuan X Bu H Zhou J. Recent advances of SHP2 inhibitors in cancer therapy: current development and clinical application. J Med Chem. 2020;63:11368–96. 32460492 10.1021/acs.jmedchem.0c00249

[36] Song Y Wang S Zhao M. Strategies targeting protein tyrosine phosphatase SHP2 for cancer therapy. J Med Chem. 2022;65:3066–79. 35157464 10.1021/acs.jmedchem.1c02008

[37] Sharma MC. Annexin A2 (ANX A2): an emerging biomarker and potential therapeutic target for aggressive cancers. Int J Cancer. 2019;144:2074–81. 30125343 10.1002/ijc.31817

[38] Chugh S Ouzounian M Lu Z. Pilot study identifying myosin heavy chain 7, desmin, insulin-like growth factor 7, and annexin A2 as circulating biomarkers of human heart failure. Proteomics. 2013;13:2324–34. 23713052 10.1002/pmic.201200455 PMC3735469

[39] Fairoozy RH Cooper J White J. Identifying low density lipoprotein cholesterol associated variants in the Annexin A2 (ANXA2) gene. Atherosclerosis. 2017;261:60–8. 28456096 10.1016/j.atherosclerosis.2017.04.010 PMC5446264

[40] Jansen M Algül S Bosman LP. Blood-based biomarkers for the prediction of hypertrophic cardiomyopathy prognosis: a systematic review and meta-analysis. ESC Heart Fail. 2022;9:3418–34. 35842920 10.1002/ehf2.14073 PMC9715795

[41] Shimada YJ Batra J Kochav SM. Difference in metabolomic response to exercise between patients with and without hypertrophic cardiomyopathy. J Cardiovasc Transl Res. 2021;14:246–55. 32594362 10.1007/s12265-020-10051-2

[42] Guo L Wang B Zhang F. Novel biomarkers identifying hypertrophic cardiomyopathy and its obstructive variant based on targeted amino acid metabolomics. Chin Med J (Engl). 2022;135:1952–61. 36156511 10.1097/CM9.0000000000002279 PMC9746752

[43] Padrón-Barthe L Villalba-Orero M Gómez-Salinero JM. Activation of serine one-carbon metabolism by calcineurin Aβ1 reduces myocardial hypertrophy and improves ventricular function. J Am Coll Cardiol. 2018;71:654–67. 29420962 10.1016/j.jacc.2017.11.067

[44] Braz JC Bueno OF De Windt LJ. PKC alpha regulates the hypertrophic growth of cardiomyocytes through extracellular signal-regulated kinase1/2 (ERK1/2). J Cell Biol. 2002;156:905–19. 11864993 10.1083/jcb.200108062 PMC2173307

[45] Marrocco V Bogomolovas J Ehler E. PKC and PKN in heart disease. J Mol Cell Cardiol. 2019;128:212–26. 30742812 10.1016/j.yjmcc.2019.01.029 PMC6408329

[46] Previs MJ O’Leary TS Morley MP. Defects in the proteome and metabolome in human hypertrophic cardiomyopathy. Circ Heart Fail. 2022;15:e009521. 35543134 10.1161/CIRCHEARTFAILURE.121.009521 PMC9708114

[47] Xu M Xue RQ Lu Y. Choline ameliorates cardiac hypertrophy by regulating metabolic remodelling and UPRmt through SIRT3-AMPK pathway. Cardiovasc Res. 2019;115:530–45. 30165480 10.1093/cvr/cvy217

[48] Sparks MA Crowley SD Gurley SB. Classical renin-angiotensin system in kidney physiology. Compr Physiol. 2014;4:1201–28. 24944035 10.1002/cphy.c130040 PMC4137912

[49] Zhou Q Wei SS Wang H. Crucial role of ROCK2-mediated phosphorylation and upregulation of FHOD3 in the pathogenesis of angiotensin II-induced cardiac hypertrophy. Hypertension. 2017;69:1070–83. 28438902 10.1161/HYPERTENSIONAHA.116.08662

[50] Okabe K Matsushima S Ikeda S. DPP (Dipeptidyl Peptidase)-4 inhibitor attenuates Ang II (Angiotensin II)-induced cardiac hypertrophy via GLP (Glucagon-Like Peptide)-1-dependent suppression of Nox (Nicotinamide Adenine Dinucleotide Phosphate Oxidase) 4-HDAC (Histone Deacetylase) 4 Pathway. Hypertension. 2020;75:991–1001. 32160098 10.1161/HYPERTENSIONAHA.119.14400

[51] Cooper RM Raphael CE Liebregts M. New developments in hypertrophic cardiomyopathy. Can J Cardiol. 2017;33:1254–65. 28941606 10.1016/j.cjca.2017.07.007

[52] Akhtar H Al Sudani H Hussein M. Effects of renin-angiotensin-aldosterone system inhibition on left ventricular hypertrophy, diastolic function, and functional status in patients with hypertrophic cardiomyopathy: a systematic review. Cureus. 2022;14:e26642. 35949750 10.7759/cureus.26642 PMC9356743

[53] van der Merwe L Cloete R Revera M. Genetic variation in angiotensin-converting enzyme 2 gene is associated with extent of left ventricular hypertrophy in hypertrophic cardiomyopathy. Hum Genet. 2008;124:57–61. 18560893 10.1007/s00439-008-0524-6 PMC2469277

[54] Philipson DJ DePasquale EC Yang EH. Emerging pharmacologic and structural therapies for hypertrophic cardiomyopathy. Heart Fail Rev. 2017;22:879–88. 28856513 10.1007/s10741-017-9648-x

[55] Meng X Cui J He G. Bcl-2 is involved in cardiac hypertrophy through PI3K-Akt pathway. Biomed Res Int. 2021;2021:6615502. 33778070 10.1155/2021/6615502 PMC7979306

[56] Chen Y Zhou J Wei Z. Identification of circular RNAs in cardiac hypertrophy and cardiac fibrosis. Front Pharmacol. 2022;13:940768. 36003513 10.3389/fphar.2022.940768 PMC9393479

[57] Wesche H Gao X Li X. IRAK-M is a novel member of the Pelle/interleukin-1 receptor-associated kinase (IRAK) family. J Biol Chem. 1999;274:19403–10. 10383454 10.1074/jbc.274.27.19403

[58] Kobayashi K Hernandez LD Galán JE. IRAK-M is a negative regulator of Toll-like receptor signaling. Cell. 2002;110:191–202. 12150927 10.1016/s0092-8674(02)00827-9

[59] Zhou H Yu M Fukuda K. IRAK-M mediates Toll-like receptor/IL-1R-induced NFκB activation and cytokine production. EMBO J. 2013;32:583–96. 23376919 10.1038/emboj.2013.2 PMC3579143

[60] Nguyen TH Turek I Meehan-Andrews T. Analysis of interleukin-1 receptor associated kinase-3 (IRAK3) function in modulating expression of inflammatory markers in cell culture models: a systematic review and meta-analysis. PLoS One. 2020;15:e0244570. 33382782 10.1371/journal.pone.0244570 PMC7774834

[61] Pai P Sukumar S. HOX genes and the NF-κB pathway: a convergence of developmental biology, inflammation and cancer biology. Biochim Biophys Acta Rev Cancer. 2020;1874:188450. 33049277 10.1016/j.bbcan.2020.188450

[62] Tang L Yao F Wang H. Inhibition of TRPC1 prevents cardiac hypertrophy via NF-κB signaling pathway in human pluripotent stem cell-derived cardiomyocytes. J Mol Cell Cardiol. 2019;126:143–54. 30423318 10.1016/j.yjmcc.2018.10.020

[63] Koivisto E Jurado Acosta A Moilanen AM. Characterization of the regulatory mechanisms of activating transcription factor 3 by hypertrophic stimuli in rat cardiomyocytes. PLoS One. 2014;9:e105168. 25136830 10.1371/journal.pone.0105168 PMC4138181

[64] Pelliccia F Limongelli G Rosano GMC. Nuclear factor-kappa B predicts long-term clinical outcome in patients with hypertrophic cardiomyopathy: 10-year follow-up study. Eur J Prev Cardiol. 2022;29:e108–11. 33760096 10.1093/eurjpc/zwab047

[65] Kuusisto J Kärjä V Sipola P. Low-grade inflammation and the phenotypic expression of myocardial fibrosis in hypertrophic cardiomyopathy. Heart. 2012;98:1007–13. 22447464 10.1136/heartjnl-2011-300960 PMC3368494

[66] Yokoyama T Nakano M Bednarczyk JL. Tumor necrosis factor-alpha provokes a hypertrophic growth response in adult cardiac myocytes. Circulation. 1997;95:1247–52. 9054856 10.1161/01.cir.95.5.1247

[67] Zhao L Cheng G Jin R. Deletion of interleukin-6 attenuates pressure overload-induced left ventricular hypertrophy and dysfunction. Circ Res. 2016;118:1918–29. 27126808 10.1161/CIRCRESAHA.116.308688 PMC4902783

